# Effectiveness of Low-Level Laser Therapy in Reducing Orthodontic Pain: A Systematic Review and Meta-Analysis

**DOI:** 10.1155/2017/8560652

**Published:** 2017-09-27

**Authors:** Naira Figueiredo Deana, Carlos Zaror, Paulo Sandoval, Nilton Alves

**Affiliations:** ^1^Master Program in Dentistry, Faculty of Dentistry, Universidad de La Frontera, Temuco, Chile; ^2^Department of Pediatric Dentistry and Orthodontics, Faculty of Dentistry, Universidad de La Frontera, Temuco, Chile; ^3^Faculty of Dentistry, Universidad San Sebastian, Puerto Montt, Chile; ^4^Applied Morphology Research Centre (CIMA), Faculty of Dentistry, Universidad de La Frontera, Temuco, Chile

## Abstract

**Objectives:**

To assess the effectiveness of low-level laser therapy (LLLT) in reducing orthodontic pain after the application of orthodontic force (OF).

**Methods:**

A systematic search was conducted in the MEDLINE, EMBASE, Scopus, Cochrane Library, Web of Science, and EBSCOhost databases. The study included randomized clinical trials (RCT) which analysed the effectiveness of LLLT in reducing orthodontic pain assessed at 24 and 72 hrs after the application of OF. The risk of bias of the eligible trials was assessed using the Cochrane Collaboration's risk of bias tool. Standard mean difference was calculated and pooled by meta-analysis using random effect models.

**Results:**

Of 467 identified articles, 20 RCT were finally included. In the risk of bias assessments, 13 studies presented a high risk, 5 an unclear risk, and 2 a low risk. The meta-analysis showed that in patients treated with laser versus placebo there was a difference in favour of LLLT in spontaneous pain 24 and 72 hrs after the installation of light archwires and spontaneous pain and chewing pain 24 and 72 hrs after the installation of elastomeric separators.

**Conclusions:**

LLLT proved to be effective in promoting a reduction in spontaneous and chewing pain after the application of OF; however, the poor quality of the evidence requires these results to be treated with caution.

## 1. Introduction

Orthodontic treatment (OT) is essential for functional and aesthetic rehabilitation of the chewing apparatus [1]. The application of orthodontic force and consequent tooth movement promote remodelling of the alveolar bone around the tooth root [2–4]. The application of force to a tooth triggers a temporary inflammatory process mediated by a variety of inflammatory cytokines, with no pathological condition [5]. The tissular response implies initial vascular changes, followed by synthesis of prostaglandins, cytokines, and growth factors which finally activate tissular remodelling, characterised by osteoclastogenesis on the pressure side and osteogenesis on the tension side [1–5]. On the compression side, bone resorption is triggered by RANK signalling, present in osteoclast precursors, and RANKL, expressed in particular by osteoblasts [6].

Orthodontic pain is an unwanted side effect; it causes great concern among patients and may be responsible for their withdrawal from OT [7]. Pain is perceived as discomfort, dull pain, and hypersensitivity in affected teeth [8]; it tends to reach its peak after 24 hours, and a reduction is observed from the third day after fitting of the brace when the tissue recovery process commences [9, 10]. The painful sensation caused by tooth movement affects the patient's quality of life and interferes in his/her chewing and speech [10]. When a mechanical force is applied to the teeth, an inflammatory reaction is triggered in the periodontal tissue [11] resulting in the release of inflammatory mediators such as prostaglandins, T substance, histamine, and serotonin [12]. Previous studies have indicated that an increase in prostaglandin-E2 (PGE2) levels is related to the initial intensity of the pain, while an increase in interleukin-1 is related to pain occurring 24 hours after the application of orthodontic force [13].

Various methods have been proposed for reducing the discomfort caused by pain during tooth movement, such as the use of anti-inflammatory medication [14], acupuncture [15], and low-level laser therapy (LLLT) [16]; however, the secondary effects of the administration of nonsteroidal anti-inflammatory drugs (NSAID) may affect the rate of movement [14, 17]. LLLT has been used as an option for treating orthodontic pain as it is easy to apply and noninvasive, and there are few contraindications or side effects [16]. Some studies have reported that LLLT is able to control pain in orthodontic patients [7, 18]; however, other studies indicate that laser cannot produce analgesia in these patients [9]. The effectiveness of laser in reducing the pain caused by orthodontic treatment is therefore still undecided. The research question for this work was therefore as follows: Are the intensity and duration of the pain produced by the application of orthodontic force lower in patients who have received near infrared low-level laser therapy than in patients who received a placebo or no therapy of any kind? The aim of this study was to assess the effectiveness of near infrared low-level laser therapy in reducing orthodontic pain after the application of orthodontic force.

## 2. Materials and Methods

### 2.1. Eligibility Criteria

A systematic review of the published data was conducted in accordance with the Cochrane Handbook for the Systematic Review of Interventions and reported according to the guidelines of the Preferred Reporting Items of Systematic Reviews and Meta-Analysis (PRISMA) [19, 20].

The inclusion criteria were as follows: (1) randomized clinical trials which analysed the effectiveness of LLLT in reducing orthodontic pain compared with a control (no treatment of any kind) and/or placebo group (simulated pain treatment); (2) participants who received orthodontic treatment with elastomeric separators, canine retraction, and/or other orthodontic treatment; (3) studies which analysed the intensity and duration of pain using the Visual Analogue Scale (VAS), Numeric Rating Scale, or another type of questionnaire; (4) studies published in English.

The exclusion criteria were as follows: (1) studies of medically compromised patients; (2) studies that used high-level laser or red laser; (3) literature reviews, in vitro studies, case or letter reports, animal studies, and unpublished theses.

### 2.2. Sources of Information and Search Strategy

A systematic search was conducted up to May 2017 in the MEDLINE, EMBASE, Scopus, Cochrane Library, Web of Science, and EBSCOhost databases. The details of the search strategy used are given in Table 1. No limit date was applied in the search for articles. The search was complemented by a manual review of the references of the studies included.

Titles and abstracts were selected independently by two investigators (N.F.D. and N.A.) to verify their eligibility. In cases of discrepancy, consensus was obtained by discussion or by consulting a third reviewer (P.S.). The references that appeared to fulfil the inclusion criteria were reviewed in full text by the same reviewers (N.F.D. and N.A.).

The data from each article selected were analysed to obtain sample size, sex, age range, laser used, wavelength, output power, spot size, number of application points, treatment time, days of LLLT application, total energy, energy density, study design, pain evaluation method, pain evaluation interval, and the principal results found for the LLLT group and the control/placebo group (event frequency, mean, and standard deviation of pain scores).

### 2.3. Assessment of Risk of Bias

Two review authors (N.F.D. and N.A.) independently assessed the risk of bias of the eligible trials according to the Cochrane Collaboration's risk of bias tool [19]. In cases of discrepancy, consensus was obtained by consulting a third reviewer (C.Z.). The domains assessed were (1) random sequence generation; (2) allocation concealment; (3) blinding of participants; (4) blinding of personnel; (5) blinding of outcome assessment; (6) incomplete outcome data; (7) selective reporting; (8) other biases (baseline imbalance, similarity in using cointerventions between groups, and inadequate statistical analysis). The potential risk of bias for each study was classified as high, unclear, or low.

### 2.4. Summary of Findings

We used the principles of the GRADE system to assess the overall quality of the body of evidence associated with the main outcomes and we constructed a “Summary of Findings” (SoF) table using the GRADEpro GDT software (http://gdt.guidelinedevelopment.org). The GRADE approach appraises the quality of a body of evidence based on the extent to which one can be confident that an estimate of effect or association reflects the item being assessed. We assessed the quality of the body of evidence with reference to the overall risk of bias of the included studies, directness of the evidence, inconsistency of the results, precision of the estimates, risk of publication bias, and magnitude of the effect [20]. Depending on the seriousness, the quality of the evidence can be downgraded by one or two levels for each aspect. We categorised the quality of the body of evidence for each of the primary outcomes as high, moderate, low, or very low.

### 2.5. Data Synthesis

The main outcome was pain assessed at 24 hrs and 72 hrs. We pooled studies that compared laser therapy with a placebo. In studies that used protocols with different irradiation doses, the protocol with the lowest doses was included in the meta-analysis. Results reported as continuous data with standard mean difference (SMD) were calculated and pooled by meta-analysis. The SMD allowed us to combine data from studies using different pain scales, such as VAS and Numeric Rating Scale [19]. For all measures, forest plots were constructed showing the summary and 95% confidence interval (CI) estimated in the meta-analyses, together with results from individual studies. We used a random effect model (DerSimonian-Laird method), as we expected variation in effects due to differences in study populations, pain scales, and methods. We combined different study designs (parallel designs and split-mouth designs) using the generic inverse variance method [19, 21].

Heterogeneity among studies was evaluated using the *I*^2^ statistical categorisation as follows: <30% not important; 30%–50% moderate; 50%–75% substantial; 75%–100% considerable [19, 21]. A subgroup analysis was performed according to type of orthodontic force used, since this could be an important source of heterogeneity. The software used was Review Manager 5.3 (Cochrane IMS, Copenhagen, Denmark).

## 3. Results

### 3.1. Study Selection

**Figure 1:**
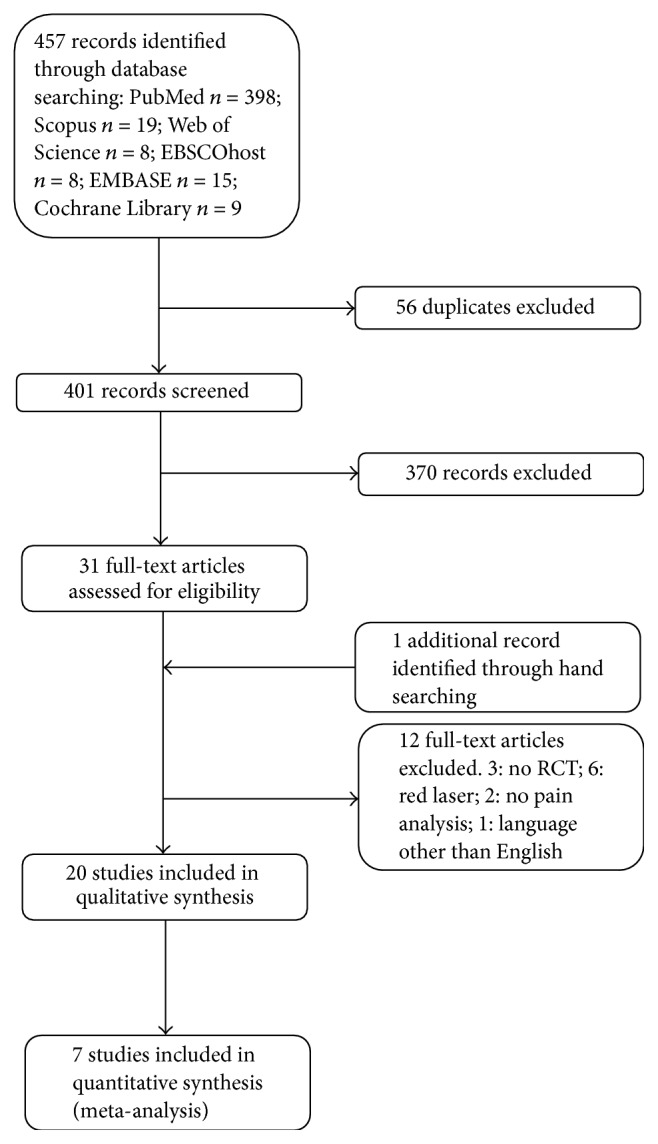
Flowchart of systematic literature review.

A flowchart of the article selection process for each stage of the review is presented in Figure 1. The search identified 457 references. After excluding duplicates and reviewing titles and abstracts, 32 articles were evaluated in full text. Subsequently, 12 potentially relevant studies were excluded and one was identified by hand search. Twenty RCT were finally included.

### 3.2. Study Characteristics

The parameters used in the studies are analysed in Table 2. We observed that in many studies the irradiation parameters were not presented; in general, however, sufficient information was given to enable them to be calculated.

#### 3.2.1. Elastomeric Separators

Twelve RCT analysed pain in patients subjected to OF with elastomeric separators [22–33]; in eight, a reduction in pain intensity was observed [22–24, 26–28, 31, 32]. Ten articles described laser application under a split-mouth design [23–26, 28–33] and two were parallel clinical trials [22, 27].

#### 3.2.2. Archwire Placement

Three studies analysed the analgesic effect of LLLT on orthodontic pain after the installation of light (initial) archwires [34–36]. All the studies reported reduced pain with the use of laser and used parallel design.

One study examined the effect of LLLT on pain in the final archwires [37]; laser was successful in reducing pain. This study used a split-mouth design.

#### 3.2.3. Canine Retraction

Four RCT assessed the effect of LLLT on orthodontic pain during canine retraction [9, 38–40]; pain reduction was observed in only one study [38]. All used split-mouth design.

#### 3.2.4. Pain Assessment and Principal Findings of Studies

The pain assessments and the main findings reported are summarised in Table 3. The Visual Analogue Scale (VAS) was the method used for pain assessment in 16 studies [9, 22, 24–34, 37, 38, 40], three studies used the Numeric Rating Scale [23, 35, 36], and one used the Wong-Baker Faces Pain Rating Scale [39]. VAS was originally proposed by Huskisson (1974) for quantifying pain. It takes the form of a line 10 cm long marked with a scale of 0 to 10 to indicate the pain level experienced, with 0 representing absence of pain and 10 intense pain [9]. Because it is easily applied and understood, VAS is used in many studies. In the articles analysed in our study, pain was measured from 5 minutes up to 120 hours after the application of orthodontic force. The pain reached a peak within 24 or 48 hours after application and reduced on the third day. Of the 20 studies included in the qualitative analysis, 13 (65%) reported finding a significant reduction in pain [22–24, 26–28, 31, 32, 34–38].

### 3.3. Risk of Bias

**Figure 2:**
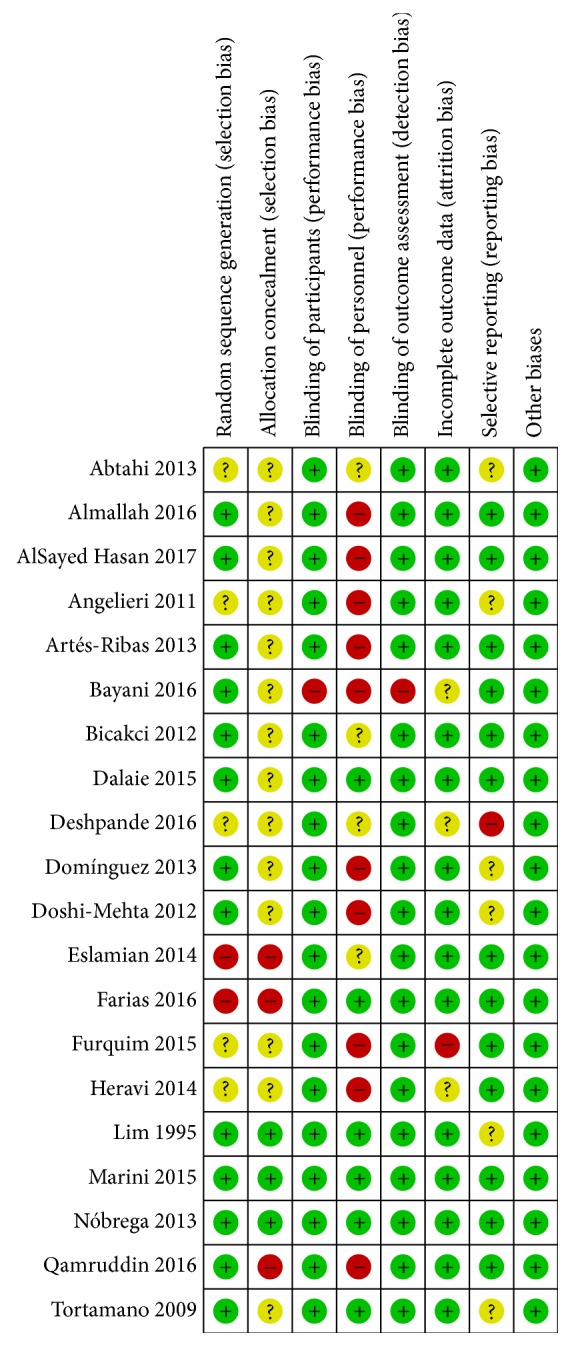
Risk of bias summary: authors' judgments about each risk of bias item for studies included.

The results of the risk of bias assessments of the studies included in this systematic review are shown in Figure 2. Of a total of 20 studies, 13 presented high risk of bias [9, 23, 24, 26, 28–30, 32, 34, 36–38, 40], five presented unclear risk [25, 31, 33, 35, 39], and two presented low risk [22, 27]. “Blinding of personnel” was the principal risk of bias observed in studies, with ten studies where the operators were not blinded [9, 23, 26, 28–30, 34, 37, 38, 40]; another four studies stated that the operators were blinded but gave no details of how this was done [31–33, 36]. Although only randomized studies were included, it was observed that two studies did not carry out “random sequence generation” correctly [24, 32], and five studies did not describe how the randomization sequence was generated [9, 29, 33, 36, 40]. Only three studies declared consistently how “allocation concealment” was done [22, 25, 27]; in three studies, the allocations could be predicted [23, 24, 32]; in one study, the authors declared that the sequence was concealed but gave no information on how the “allocation concealment” was effected [30], and the other studies did not offer sufficient information to judge the concealment of the randomization sequence. “Blinding of participants” and “blinding of outcome” were carried out in all studies except one [34], in which the authors declared that blinding was impossible due to the different pain management approaches employed in their study. One study presented “incomplete outcome data” due to the withdrawal of a large number of participants, especially in one of the groups [29]; two studies gave no information on losses [36, 40] and one declared only 10% losses but did not state which groups lost participants, making it impossible to assess whether the lack of patient follow-up had any impact on the results [34]. Six studies did not present sufficient information to judge “selective reporting” [9, 25, 33, 35, 37, 38] and one study did not report all the results declared in the methodology [36].

### 3.4. Effectiveness of LLLT (Near Infrared) in Reducing Orthodontic Pain

#### 3.4.1. Spontaneous Pain

**Figure 3:**
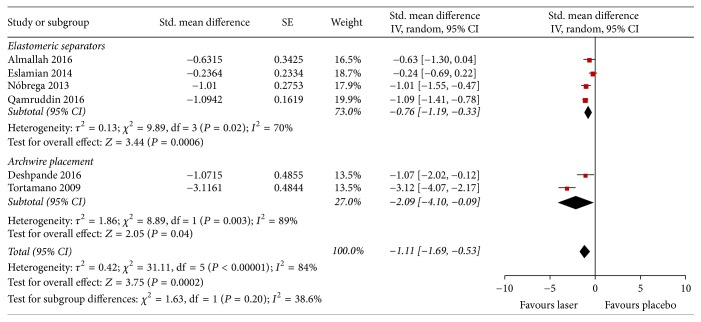
Forest plot of pooled standard mean difference in spontaneous pain at 24 hours.

Six studies reported sufficient data to assess the intensity of spontaneous pain after 24 hrs; these were divided into two subgroups according to the different types of orthodontic force (Figure 3). In the analysis of spontaneous pain 24 hrs after the installation of elastomeric separators, four studies with low quality of evidence were compared, of which three assessed pain by VAS and one by Numeric Rating Scale. Less intensity of pain was observed in patients treated with laser (near infrared) than in those with placebo (SMD −0.76; 95% CI −1.19 to −0.33; *I*^2^ = 70%). The comparison of spontaneous pain 24 hrs after the installation of light archwires between patients treated with laser versus placebo showed a difference in favour of LLLT (SMD −2.09; 95% CI −4.10 to −0.09; *I*^2^ = 89%). The quality of evidence was also judged to be low, meaning that we had low confidence in the estimate of effect. The overall assessment was significantly in favour of LLLT (near infrared) (SMD −1.11; 95% CI −1.69 to −0.53; *I*^2^ = 38.6%).

**Figure 4:**
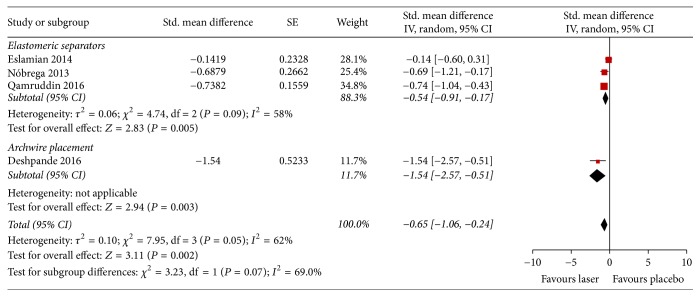
Forest plot of pooled standard mean difference in spontaneous pain at 72 hours.

The findings for spontaneous pain after 72 hrs were compared in four studies (Figure 4); two used the Numeric Rating Scale and two used VAS. Three studies with low quality of evidence presented sufficient data for analysing spontaneous pain after the installation of elastomeric separators: a significant reduction in pain intensity was observed in the groups treated with laser (near infrared) as compared to placebo (SMD −0.54; 95% CI −0.91 to −0.17; *I*^2^ = 58%). One study with low quality of evidence compared the spontaneous pain 72 hrs after the installation of light archwires in patients treated with laser versus no treatment: the pain intensity was lower in patients treated with laser than in patients with no treatment (SMD −1.54; 95% CI −2.57 to −0.51). In the overall assessment, a significant reduction in pain intensity was observed in the laser-irradiated group compared with the placebo group (SMD −0.65; 95% CI −1.06 to −0.24; *I*^2^ = 69%).

#### 3.4.2. Chewing Pain

**Figure 5:**
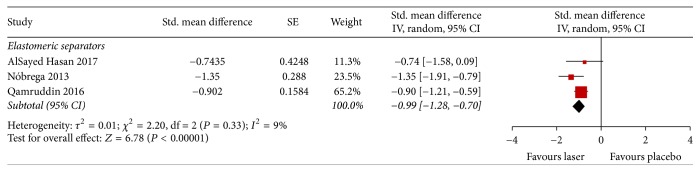
Forest plot of pooled standard mean difference in chewing pain at 24 hours.

**Figure 6:**
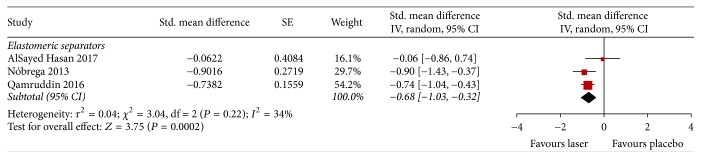
Forest plot of pooled standard mean difference in chewing pain at 72 hours.

Three studies assessed pain intensity during chewing 24 and 72 hrs after the installation of elastomeric separators (Figures 5 and 6). Two of these used VAS and one used the Numeric Rating Scale. The intensity of chewing pain 24 hrs after the installation of elastomeric separators was less in the laser-treated group than the placebo group (SMD −0.99; 95% CI −1.28 to −0.70; *I*^2^ = 9%); a similar pattern was observed after 72 hrs (SMD −0.68; 95% CI −1.03 to −0.32; *I*^2^ = 34%). For both outcomes the quality of evidence was judged to be low, meaning further research is very likely to have an important impact on our confidence in the estimate of effect.

#### 3.4.3. Quality of Evidence Summary

All the studies included were randomized controlled trials. However, methodological issues limited the overall quality of evidence. We downgraded their quality mainly due to the high risk of bias associated with selection bias, performance bias, and selective report bias. Moreover, the low number of participants for some outcomes led to additional downgrading for imprecision of the effect estimate. Selection bias was judged as high risk of bias due to the use of inadequate methods to generate the random sequence and lack of allocation concealment. The performance bias was downgraded in some studies because it was not possible to blind the personnel; however, all studies included in the analysis reported that the outcome assessor was blinded. Only one study presented selective reporting. Our final assessment was that all the outcomes presented low quality of evidence (Table 4).

## 4. Discussion

### 4.1. Summary of the Evidence

Tooth movement is dependent on a painful, inflammatory adaptation of the alveolar process [17]. The pain caused by tooth movement is a constant concern among patients. Pain perception varies considerably from patient to patient; it is a highly subjective sensation and consequently very difficult to quantify in scientific investigation [41].

Due to the inflammatory nature of orthodontic pain, nonsteroidal anti-inflammatory drugs (NSAID) have been considered the gold standard for controlling pain in orthodontic patients [4], administered when the patient suffers unbearable pain. NSAID inhibits the synthesis of prostaglandins, which are important mediators of pain induction [4]. It should be noted that the use of these drugs is associated with gastrointestinal problems, thrombocytopenia, skin rashes, renal insufficiency, hypertension, and headaches [8]. LLLT presents no serious side effects such as are related to NSAID [22]; furthermore, some studies have shown that LLLT is effective not only in reducing orthodontic pain but also in increasing the rate of tooth movement in canine retraction [7, 38, 42]. Domínguez-Camacho and Velasquez-Cujar [43] indicate that LLLT reduces the average time of treatment by 30% and is effective in accelerating dental movement not just in a specific phase of treatment. In a randomized clinical trial, Bayani et al. [34] compared the effects of ibuprofen, low-level red laser (660 nm), low-level infrared laser (810 nm), and bite wafers in orthodontic pain management. These authors report that low-level infrared laser (810 nm) was the most effective strategy for pain relief following initial wire installation and can be considered an alternative to ibuprofen. LLLT promotes local effects on inflammation less than 24 hours after irradiation, as well as reducing levels of PGE2, tumour necrosis factor, plasminogen activator, and COX-2 expression [10]. One of the mechanisms by which laser reduces pain is by producing an alteration in the conduction of action potentials in the peripheral nerves through the generation of varicosities which reduce the speed of fast axonal flow and reduce the mitochondrial membrane potentials, resulting in reduced availability of ATP and neurotransmission failure in A*δ* and C nociceptor fibres [44]. According to Montesinos [45], another way in which pain reduction is promoted is through stimulation of beta-endorphin production, a natural mediator produced by the organism which reduces pain. LLLT also inhibits the release of arachidonic acid, which acts on damaged cells to generate metabolites which interact with pain receptors [46].

When using laser, it is important to choose the most appropriate wavelength for each disease [47]. Laser penetration of the tissues is directly related to wavelength [48]. A wavelength of 830 nm presents the deepest penetration, able to reach the cortical and alveolar bone tissues; it is more effective than wavelengths between 620 and 670 nm [49]. Because red and infrared laser are indicated in different situations [47], we consider it important to analyse their effects separately; in the present investigation, therefore, we only considered RCT with low-level laser (near infrared) at wavelengths between 780 nm and 940 nm. Red laser has weaker penetration, mainly due to the absorption mechanism by which it interacts with biological tissue; it is therefore indicated for superficial lesions, such as tissue repair (healing and local drainage). Infrared laser by contrast achieves deeper penetration due to its interaction through changes of polarity in the biomembranes. Because of its wavelength, infrared laser has been the treatment of choice for promoting immediate and temporary analgesia, acting on the cell membrane to cause hyperpolarization, that is, a photo-physical change as a result of the light-cell biological interaction [47]. Endorphin synthesis and the action potential of neural cells increase, whereas the amount of bradykinin as well as the activity of the C-fibres driving the pain stimuli decreases [50], resulting in relief of painful symptoms [46]. Of the 13 studies which found pain reduction with LLLT, ten used a wavelength between 800 and 830 nm and three between 910 and 940 nm. In the seven studies in which no pain reduction was observed, five used wavelengths between 780 and 830 nm and two used wavelengths between 880 nm and 940 nm. Although the results are not unanimous, studies which used wavelengths between 800 and 830 nm reported a greater analgesic capacity than studies which used 904–940 nm, corroborating the findings of Yamaguchi et al. [51]. Some researchers report that when pulsed mode is used, multiple photo-dissociation events can occur, promoting greater penetration by the laser light than in continuous mode where the number of dissociations may be much smaller [52]. Some studies reveal that pulsed light promotes better tissue repair and reduces the behavioural manifestations of somatic pain when compared with continuous wave [53, 54]. According to Antczak-Bouckoms et al. [55], the decision to choose split-mouth design will depend on the nature of the disease and the effect of the treatment. Some authors state that split-mouth design allows better pain evaluation since it eliminates interindividual variation resulting from sex, age, and pain perception [23]. A great advantage of split-mouth design is the smaller sample size required compared to parallel studies, since each patient acts as his own control [56]. When split-mouth design is used, the intervention sites in each patient must be uniform; this is not usually a problem in orthodontics, since intact dentitions are more often available, meaning that comparable sites are more feasible [57]. It must be noted that the lack of uniformity between sites in participants may introduce a selection bias, since interventions may be applied in sites with different baseline characteristics [57]. Another advantage of studies with split-mouth design is that the loss or withdrawal of participants does not create an imbalance between groups for analysis of the results; however, the loss/withdrawal rate cannot be so high that it affects the result of the study. One possible disadvantage of split-mouth as compared to parallel design in studies using laser irradiation is that operator blinding is more difficult, since the laser and the simulation (placebo) are generally applied in the same session, and this introduces a bias into the study. In our investigation, we observed that 15 studies (75%) used split-mouth design; of these, only three reported a double-blind study [24, 25, 39].

Only two studies had low risk of bias [22, 27]; five studies presented an unclear risk [25, 31, 33, 35, 39] and the other 13 presented a high risk [9, 23, 24, 26, 28–30, 32, 34, 36–38, 40]. “Allocation concealment” and “blinding of personnel” were the main weaknesses in study execution, and only 30% reported correct application of this precaution [22, 24, 25, 27, 35, 39]. The fact that the majority of studies presented a high or unclear risk of bias means that the results must be interpreted with caution.

LLLT single application proved effective for pain reduction in ten studies [22–24, 26–28, 34–37], while in three studies pain was reduced with two applications of LLLT [28, 31, 32], and in one study LLLT was effective after four applications [38]. Almallah et al. [28] carried out a study comparing single dose with double dose and found no differences in pain reduction. We observed that there are no studies in the literature reporting the “ideal” number of LLLT applications; however, we can say that a single dose after the application of OF proved sufficient to reduce pain.

The pain evaluation method used in 80% of the studies was VAS. According to Farias et al. [24] and Bicakci et al. [31], in patients treated with LLLT, significant pain reduction was observed 24 hours after the application of orthodontic force. Orthodontic pain begins two hours after orthodontic activation [35] and tends to be more severe after 6–24 hours [23, 26, 28–30, 32–34, 36, 37]; it presents a reduction after two days [26], three days [27, 35, 36], or five days [24, 33]. According to the literature, pain is more intense in patients in the control/placebo group [22–24, 26–28, 31, 32, 34, 35, 37, 38]. Oral pain is less intense in patients treated with LLLT [35]. Marini et al. [22] indicate that in the LLLT group the score on the VAS scale was always close to zero, with a maximum of 3.5, while the control group presented a minimum of 3.0 shortly after activation and a maximum of 6.5 at 36 hours after orthodontic activation. The pain intensity described by orthodontics patients varied according to the type of OF applied. When elastomeric separators were used, the highest spontaneous pain measured by VAS (0–10) after 24 hrs was 5.25 for the LLLT group [33], 4.71 for the placebo group [24], and 6.1 for the control group [22]. The highest pain level found in participants who used elastomeric separators was reported by Abtahi et al. [33] for the placebo group 48 hrs after application of OF, with a mean value of 7.45. The highest intensity reported by Nóbrega et al. [27] was 6.45. In canine retraction, the pain in the experimental and placebo groups was similar in all periods [9, 38]. According to the literature, the highest levels of pain were found after the installation of light archwires, with a value of 8.55 for the placebo group 24 hrs after the application of OF, measured using the Numeric Rating Scale [35]. Bayani et al. [34] state that patients in the placebo group reported chewing pain of up to 7 on the VAS, while patients in the experimental group (treated with laser) reported an average value of 3.2. The authors also report that pain lasted for longer in the untreated group [22, 24, 27] and took longer to disappear, persisting in 10% of subjects in the laser group and in 70–80% of subjects in the control/placebo group [22]. In Nóbrega et al. [27], the patients in both the control and irradiated groups stated that pain in occlusion was more severe than spontaneous pain, hindering chewing. These authors also observed that an expressive percentage of patients in the placebo group, up to 60%, still presented occlusion pain on day 5 after activation, while only 23.3% of the patients in the LLLT group still presented occlusion pain [27].

In the present meta-analysis, 24 and 72 hrs after the installation of elastomeric separators the LLLT group presented lower mean values for spontaneous and chewing pain than the placebo group. In patients fitted with light archwires, laser was effective in reducing spontaneous pain at 24 and 72 hrs after OT. Patients treated with laser reported less pain intensity (spontaneous and chewing) 24 hrs after OT, when the peak pain usually occurs [23, 26, 29, 32–34, 37]. Laser also proved to have a prolonged analgesic effect, reducing pain even 72 hrs after the installation of elastomeric separators. It should be noted that methodological issues limited the overall quality of evidence. The studies presented risk of bias associated with selection bias, performance bias, and selective reporting bias, compromising the internal validity of the investigation, since all the studies presented low quality of evidence.

Bjordal et al. [10] observed that optimal effects of LLLT on acute pain can be achieved by using a dose of 7.5 J/cm^2^ in the first 72 hours after the injury to reduce inflammation; the dose must be reduced in subsequent days, typically to 2 J/cm^2^, to promote tissue repair. Lizarelli [47] indicated a dose of ≥5 and <20 J/cm^2^ for severe pain so as not to inhibit cell activity. Other authors state that higher doses, for example, 35 J/cm^2^ [47], are needed to reduce orthodontic pain and that doses of 5 J/cm^2^ are not effective [9]. We observed that studies with similar protocols reported conflicting results. Farias et al. [24], Furquim et al. [29], and Abtahi et al. [33] used the same total energy (6 J/tooth); moreover, Farias et al. [24] and Furquim et al. [29] used similar wavelengths; however, only Farias et al. [24] reported a reduction in pain. We also observed that different irradiation parameters promoted analgesia in orthodontic patients. Qamruddin et al. [23] and Artés-Ribas et al. [26] used the same total energy (12 J/tooth) but different wavelengths, 940 nm and 830 nm, respectively; both reported successful treatment. Artés-Ribas et al. [26] and Bicakci et al. [31], who were also successful in reducing orthodontic pain, used similar wavelengths and doses (J/cm^2^/tooth); however, they used different total energies, of 12 J and 1 J, respectively. When we analysed the parameters used in the studies in which no reduction in orthodontic pain was found, we observed that they used a wavelength outside the range 808–830 nm, and/or the total energy administered was very high. Based on the above, we agree with studies which state that the success of LLLT is related to the energy applied [9, 47, 58]. We also think that energy density (J/cm^2^/tooth) cannot be the only parameter determining successful treatment. Moreover, laser therapy appears to present better results when it is associated with the use of a wavelength of 800–830 nm and total energy ≤12 J/tooth/treatment session; energy density is highly variable, with pain reduction being achieved with applications ranging from 5 J/cm^2^ to 160 J/cm^2^ per tooth. The laser irradiation parameters must be selected according to the clinical situation, based on the current phase of the lesion and considering the optical characteristics of the tissue to be irradiated and the laser irradiation methodology (point or sweep, contact or noncontact) [47]. The area to be irradiated is determined by the type of device used to apply the laser; the dose applied to the tissue can be changed by changing the spot size. Thus, the energy density applied to the tissue can be increased by reducing the spot size, which will also result in greater irradiance and penetration of the laser into the biological tissue [47]. In this meta-analysis, when more than one protocol was reported by any author, the protocol which used the lowest energy dose was selected.

More than 50% of the studies analysed present an incomplete or unclear irradiation protocol, failing to include important information such as spot size, energy density per spot and per tooth, application time/spot, and total energy per spot and per tooth. Although the studies often presented sufficient information for missing parameters to be calculated, the absence of these data complicates routine use of the protocols, often preventing reproduction in clinical practice. Future studies should seek to improve the methodological criteria used in order to allow comparison between all the parameters used by each author. New double-blind, randomized clinical trials reporting the correct allocation of patients, complete information on the protocol used, the application method, and the use of one group of patients for the control and another group to form the LLLT group, with well-defined inclusion/exclusion criteria, would reduce the risk of bias which arises during research activities and analysis of the results.

In the present investigation, 13 studies (65%) reported pain reduction in orthodontic patients with the use of LLLT. Note that in seven studies (35%) no differences were found in pain intensity between patients in the LLLT group and those who did not receive LLLT; these were three studies of canine retraction [9, 39, 40] and four in which elastomeric separators were fitted [25, 29, 30, 33]. AlSayed Hasan et al. [30] compared two different doses of LLLT, one of 2.25 J/cm^2^/tooth and one of 9 J/cm^2^/tooth; neither of the protocols proved effective in reducing pain after the fitting of elastomeric separators. Lim et al. [25] compared three different protocols with application times of 15 s, 30 s, and 60 s per tooth; all three protocols failed to reduce orthodontic pain caused by the fitting of elastomeric separators. Hawkins and Abrahamse [59] state that dosage (or fluence) can alter cell processes. The application of a low or very low dose may produce no effect, while very high doses may produce negative or inhibiting effects. This may explain why studies were found with contradictory results [60]. The origin of contradictory scientific evidence may be related to the multiple methods used for laser irradiation, as there is no protocol indicating which irradiation doses are most effective. There is a need for new studies presenting a low risk of bias to discover which laser irradiation protocol offers the greatest analgesic power in orthodontic patients.

We agree with Marini et al. [22] when they suggest the possibility of using the LLLT protocol in daily orthodontic practice. New studies are needed to develop a complete protocol for easy application and execution in clinical practice, in order to convert safe laser irradiation with effective dosing into a routine treatment for orthodontic pain.

### 4.2. Study Limitations

We identified some limitations in our review process. First, there is the possibility that we failed to identify all studies because we only considered articles published in English. However, we believe that this was minimized due to the large number of databases used, the additional search of references by hand, and the double independent review process used. Second, some studies could not be included in any of the meta-analyses because of the lack of the specific estimator needed; however, their individual data were consistent with our findings. Third, the internal validity of the summary provided by a meta-analysis depends on the quality of the primary studies; the risk of bias in the most of studies included was high. Finally, because we did not have more than ten studies to pool in any meta-analysis, funnel plots to explore possible publication biases were not constructed.

## 5. Conclusions

Randomized clinical trials to assess the effect of laser (near infrared) on orthodontic pain present great heterogeneity of irradiation parameters; conflicting results were found even in studies using similar parameters. The heterogeneity of LLLT protocols for the treatment of orthodontic pain hinders comparison between studies; moreover, it has not yet been possible to standardise the best protocol for routine use in clinical practice. New studies are needed to establish an effective LLLT protocol to obtain greater analgesia in patients undergoing orthodontic forces, which can be used routinely in clinical practice. The majority of the RCT in the present study reported results which favoured laser, showing that LLLT is beneficial for the patient; however, there were an expressive number of studies reporting that laser was not effective. LLLT has been shown to be effective in reducing spontaneous and chewing pain after the installation of elastomeric separators and light archwires. Furthermore, the analgesic effect of laser extends for 72 hrs after the installation of elastomeric separators, reducing spontaneous and chewing pain; however, the poor quality of the evidence requires these results to be treated with caution.

## Figures and Tables

**Table 1:** Search strategy and results for PubMed, EMBASE, Cochrane Library, Web of Science, Scopus, and EBSCOhost.

Database	Search strategy	Results
MEDLINE/PubMed	# 1: Low-level light therapy [Mesh] OR Low level laser therapy (Pubmed) OR Laser therapy (Pubmed)	76,795
	# 2: Analgesia [Mesh] OR Discomfort (Pubmed)	76,088
	# 3: Orthodontic^*∗*^ (Pubmed)	62,754
	# 4: # 1 AND # 2 AND # 3	398

EMBASE	# 1: Low-level laser therapy	16,792
	# 2: Pain AND tooth pain	6,559
	# 3: Orthodontics	60,423
	# 4: controlled clinical trial	597,953
	# 5: # 1 AND # 2 AND # 3 AND # 4	15

Cochrane Library	# 1: (Low-level light therapy)	366
	# 2: (Pain)	27,139
	# 3: (Orthodontics OR Orthodontic appliances, OR Orthodontic Anchorage procedures)	54
	# 4: # 1 AND # 2 AND # 3	9

Web of Science	# 1. low-level laser therapy AND Clinical trial AND Pain AND Orthodontics	8

Scopus	# l. low-level laser therapy AND Clinical trial AND Pain AND Orthodontics	19

EBSCOhost	## 1: Low-level laser therapy OR Laser therapy OR LLLT	612
	# 2: Pain	13,209
	# 3: Orthodontic treatment	6,779
	# 4: # 1 AND # 2 AND # 3	8

**Table 2:** Summary of laser irradiation parameters used in each study.

Authors	Subjects M : F	Age	Type of laser/*λ*/mode	SP	ED (J/cm^2^)	OP (mW)	Points	*t*	Frequency of laser therapy	TE (J)	Study design	LLLT is effective in reducing pain
*Elastomeric separators*												

Abtahi et al. [33]	29/24 : 5	12–22	GaAs 904 nm, pulsed mode	0.38 cm^2^	NI	200 mW	4 points: (cervical and radicular) 2 vestibular 2 lingual	30 s	0, 1, 2, 3, and 4	1.5/point 6/tooth	Single blind, split-mouth	No

Almallah et al. [28]	36	12–26	GaAlAs 830 nm	7 mm	16/tooth	100 mW	8 lingual, 8 palatal	28 s/area	Single dose and 0, 1	NI	Single blind, split-mouth	Yes

AlSayed Hasan et al. [30]	13/3 : 9	Mean age 18.16	GaAlAs 830 nm, continuous mode	NI	2.25/tooth	150 mW	2 points: mesial and distal, cervical third	15 s/point	Single dose	2/point 4/tooth	Single blind, split-mouth	No
	13/4 : 7	Mean age 18.30	GaAlAs 830 nm, continuous mode	NI	9/tooth	150 mW	2 points: mesial and distal, cervical third	60 s/point	Single dose	8/point 16/tooth	Single blind, split-mouth	No

Artés-Ribas et al. [26]	20/06 : 14	19–33.8	GaAlAs 830 nm continuous mode	0.4 cm^2^	5/point 30/tooth	100 mW	6: 3 vestibular, 3 lingual	120 s/tooth	Single dose	2/point, 12/tooth	Single blind, split-mouth	Yes

Bicakci et al. [31]	19/08 : 11	13.5–14.5	GaAlAs 820 nm	0.0314 cm^2^	7.96/point 31.84/tooth	50 mW	4: mesiobuccal mesiopalatal distobuccal distopalatal	5 s/point	0, 24 h	0.25/point 1/tooth	Single blind, split-mouth	Yes

Eslamian et al. [32]	37/12 : 25	11–32	GaAlAs 810 nm, continuous mode	NI	2/point	100 mW	10: 5 buccal, 5 palatal	20 s/point	0, 24 h	NI	Single blind, split-mouth	Yes

Farias et al. [24]	30	18–40	GaAlAs 810 nm	0.028 cm^2^	2/point 6/total	100 mW	3 points: interdental papilla, distal, near the apex	15 s/point	Single dose	6/tooth	Double blind, split-mouth	Yes

Furquim et al. [29]	79	13–34	GaAlAs 808 nm	NI	80/tooth	NI	NI	NI	Single dose	6/tooth	Single blind, split-mouth	No

Lim et al. [25]	39	21–24	GaAlAs 830 nm	NI	NI	30 mW	1: middle third	15 s; 30 s; 60 s/tooth	0, 1, 2, 3, 4, and 5	0.45; 0.95; 1.8	Double blind, split-mouth	No

Marini et al. [22]	120/64 : 56	20–25	GaAs 910 nm superpulsed mode	0.5 cm^2^	NI	160 mW	2: cervical third (buccal and lingual)	340 s/total	Single dose	54.4/total energy	Double blind, parallel	Yes

Nóbrega et al. [27]	60/22 : 38	12–26	GaAlAs 830 nm	2 mm	1/point 5/total	40.6 mW	4 points (vestibular)	25/point 125 s total	Single dose	5/tooth	Double blind, parallel	Yes

Qamruddin et al. [23]	88/28 : 60	13–30	GaAlAs 940 nm, continuous mode	NI	NI	200 mW	3 points buccally: mesial, distal middle	20 s/point	Single dose	4/point 12/tooth	Single blind, split-mouth	Yes

*Archwire placement (initial stage)*												

Bayani et al. [34]	40	14–21	GaAlAs 810 nm	0,28	3,6/tooth	200 mW	6: cervical, middle, apical (vestibular and lingual)	30 s/tooth	Single dose	6/tooth	Parallel	Yes

Deshpande et al. [36]	30	16–25	GaAs 904 nm	5 mm	NI	10 W	Lingual and buccal: middle third region	120 s/side	Single dose	NI	Single blind, parallel	Yes

Tortamano et al. [35]	60/18 : 42	12–18	GaAlAs 830 nm	NI	5/tooth	30 mW	10: 2 apical, 1 middle third, 2 cervical (palatal, lingual)	16/point	Single dose	NI	Double blind, parallel	Yes

*Archwire placement (final stage)*												

Domínguez and Velásquez [37]	59/19 : 40	NI	GaAlAs 830 nm	600 *μ*m	80/point	100 mW	2: 1 vestibular, 1 palatal	44 s/tooth	Single dose	4.4/tooth	Single blind, split-mouth	Yes

*Canine retraction*												

Angelieri et al. [9]	12	Mean age 12.66	ArGaAl 780 nm	NI	5/point	20 mW	10 points: 5 buccal and 5 lingual	10 s/point	0, 3, and 7	0.2/point 2/tooth	Single blind, split-mouth	No

Dalaie et al. [39]	12/3 : 9	Mean age 20.1	GaAlAs 880 nm, continuous mode	NI	5/point	100 mW	8 points: 4 buccally 4 lingually	10 s/point	1, 3, 7, 30, 33, 37, 60, 63, and 67	NI	Double blind, split-mouth	No

Doshi-Mehta and Bhad-Patil [38]	20/8 : 12	12–23	GaAlAs 800 nm, continuous mode	NI	5/total	0.7 mW	2: 1 buccal and 1 lingual	10 s/point	0, 3, 7, and 14 (first month and thereafter every 15 days)	8/tooth	Single blind, split-mouth	Yes

Heravi et al. [40]	20/03 : 17	15–31	GaAlAS 810 nm, continuous mode	0.28 cm^2^	21.4/point	200 mW	10 points: 5 buccal and 5 lingual	30 s/point	0, 4, 7, 11, 15, 32, 35, 39, 43, and 56	6/point 60/tooth	Single blind, split-mouth	No

M: male, F: female, *λ*: wavelength, OP: output power, *t*: time, TE: total energy, ED: energy density, SP: spot size, OT: orthodontic treatment, and NI: not informed.

**Table 3:** Description of the principal findings for the control group and the group irradiated with LLLT, with pain assessment method and interval after orthodontic activation.

Author	*Evaluation method*	*Evaluation interval after start of orthodontic treatment*	*Outcomes*	Evaluation of pain level	
				Laser group (LG)	Control group (CG)/placebo
Abtahi et al. [33]	VAS	Pain levels were measured for 5 days	No statistically significant difference between the placebo and experimental groups	(1) Days 1, 3, 4, and 5: no statistically significant difference between groups. (2) Day 2: pain level significantly lower in LLLT group (*P* = 0.009).	(1) The maximum level of pain was recorded 1 day after placement of separators and gradually declined until day 5.

Almallah et al. [28]	VAS	1, 6, 24, 48, and 96 hours	LLLT reduced the orthodontic pain caused by elastomeric separators	(1) Peak pain occurred after 24 hrs in the group which received a single irradiation and after 48 hrs in the group which received double irradiation. (2) No differences were found between pain intensity with single or double irradiation.	

AlSayed Hasan et al. [30]	VAS	1, 6, 12, 24, 48, and 72 hours	A single application of LLLT was not effective in reducing pain caused by elastomeric separators	(1) Peak pain occurred 24 hrs after application of orthodontic force in both the placebo group and the experimental group. (2) Both LLLT protocols (4 J and 16 J) failed to reduce pain.	

Angelieri et al. [9]	VAS	12, 24, 48, and 72 h after LLLT Repeated in the 2nd month	The laser protocol used in the study was not effective in reducing pain sensitivity	(1) No statistically significant difference in pain reduction was found between the irradiated and the control sides.	

Artés-Ribas et al. [26]	VAS	5 m, 6 h, 24 h, 48 h, and 72 h	The LLLT parameters used can reduce pain in patients following placement of orthodontic rubber separators	(1) Significant pain reduction as compared with the control/placebo side (*P* = 0.0001).	(1) Peak pain in 24 hours. (2) Pain reached its peak after 6–24 hours and decreased thereafter between 48 and 72 h.

Bayani et al. [34]	VAS	2 h, 6 h, bedtime, 24 h, 2, 3, and 7 days	A single irradiation of LLLT was effective in reducing pain following initial archwire placement	(1) Peak pain occurred 24 hrs after application of orthodontic force. (2) Low power red laser could not be recommended for pain control following placement of orthodontic appliances. (3) VAS presented similar mean values to those in patients treated with ibuprofen, LLLT, and bite wafer.	

Bicakci et al. [31]	VAS	5 m, 1 h, 24 h	Significant reduction in pain and PGE_2_ after 24 hours in the group irradiated with LLL	(1) Significant pain reduction was observed with laser treatment 24 h after application. Significant differences in pain level were observed between LG and CG after 24 h (*P* = 0.001). (2) The mean PGE_2_ levels showed a gradual decrease. (3) Significant differences in pain level were observed between LG and CG after 1 and 24 h (*P* = 0.001 and *P* = 0.001, resp.)	(1) The mean PGE_2_ levels were significantly elevated.

Dalaie et al. [39]	Wong-Baker Faces Pain Rating Scale was utilized	Days 1, 33, and 63	No solid evidence was found to support the effectiveness of laser for pain reduction	(1) There was no statistically significant difference in pain reduction between LG and CG.	

Deshpande et al. [36]	Scale used by Tortamano et al. modified (Harazaki + Number Rating Scale)	1, 24, 48, and 72 h after LLLT	LLLT reduced pain duration and intensity fixed orthodontic therapy patients	(1) Peak pain occurred 1 day after application of orthodontic force. (2) The pain disappeared 3 days after application of orthodontic force.	(1) Peak pain occurred 2 days after application of orthodontic force. (2) The pain disappeared 3 days after application of orthodontic force.

Domínguez and Velásquez [37]	VAS	2 h, 6 h, and 24 h Days: 2, 3, and 7	LLLT is effective for pain reduction throughout orthodontic treatment	(1) Pain was significantly less in the LLLT group at all the intervals measured (*P* < 0.00001). (2) Peak pain in 24 hours.	

Doshi-Mehta and Bhad-Patil [38]	VAS	Days: 1, 3, and 30	Pain level reduced significantly with the use of LLLT	(1) Significant pain reduction on days 3 and 30 as compared with the first day (*P* = 0.0001 and *P* = 0.0000, resp.). (2) Significant pain reduction in the irradiated group as compared with the CG on days 3 and 30 (*P* = 0.0000, *P* = 0.0000, resp.).	(1) Significant pain reduction after 30 days as compared with day 1 (*P* = 0.0016).

Eslamian et al. [32]	VAS	6 h, 24 h, and 30 h Days: 3, 4, 5, 6, and 7	LLLT reduced pain perception in the first 3 days after orthodontic separation	(1) After 6, 24, and 30 h and on day 3 the pain was significantly less in LG (*P* = 0.031, *P* = 0.014, *P* = 0.043, and *P* = 0.047, resp.). (2) Pain intensity peaked at 6 and 30 h after placing elastomeric separators. (3) Greatest pain was recorded in the mandible at 24 and 30 h.	(1) Pain intensity peaked between 6 and 30 h after placing elastomeric separators. (2) Greatest pain was recorded in the mandible at 24 h.

Farias et al. [24]	VAS	5 min., 24 and 120 hours	AlGaAs diode LLLT (810 nm) is an effective therapeutic method to control or reduce pain in the early stages of orthodontic treatment	(1) After 24 h a 13.89% reduction in pain was promoted. (2) LLLT (810 nm) was effective in pain reduction from the first 24 hours up to the fifth day (120 hours) after separator placement. (3) Lower pain intensity was recorded in the group treated with LLL after 5 minutes, 24 hours, and 120 hours.	(1) After 24 h there was a 44.39% increase in pain. (2) A significant pain reduction was observed 120 hours after installation of elastomeric separators.

Furquim et al. [29]	VAS	6 h, 12 h Days: 1, 2, and 3	LLLT did not produce significant effects on the perception of pain caused by orthodontic separation	(1) The pain peak perceived by patients occurred between 12 hours and 1 day.	

Heravi et al. [40]	VAS	Days 0, 4, 7, 11, 15, 28, 32, 35, 39, 43, and 56	LLLT with the parameters used in this studio did not influence the pain perceived by the patients	(1) No significant difference was found in VAS scores between the laser and the placebo groups.	

Lim et al. [25]	VAS	Before and after LLLT for 5 days	No statistically significant difference between the placebo and experimental groups	(1) Pain intensity was lower in the experimental group than in the placebo group; however, no statistically significant difference was observed.	

Marini et al. [22]	VAS	0, 12, 24, 36, 48, 72, and 96 h	LLLT is effective in reducing pain intensity and duration	(1) No significant difference between the arches. (2) Statistically significant differences between groups at 12, 24, 36, 48, 72, and 96 h (*P* = 0.0001); the mean VAS values of the laser groups were lower than placebo and control groups. (3) The pain never disappeared in 10% of subjects in the laser group, 70% of subjects in the placebo group, and 80% of subjects in the CG. (4) No significant differences were found between the placebo and CG. (5) The intensity and duration of pain were lower in the LG than in the placebo and CG. (6) The intensity of pain was lower in the placebo group than the CG.	

Nóbrega et al. [27]	VAS	2, 6, 24, 72, 120 hours	Laser irradiation controlled the original pain when the elastomeric separators were fitted	(1) The levels of spontaneous and occlusion pain were significantly lower in patients treated with LLL as compared with the placebo group at 2, 6, and 24 hours, and 3 and 5 days. (2) Two hours after installation of the elastomeric separators, 10% of the patients presented spontaneous pain and 16.7% occlusion pain. After 24 hours, 50% of the patients presented spontaneous pain and 66.7% occlusion pain. After 5 days, 10% of the patients presented spontaneous pain and 23.3% occlusion pain.	(1) Two hours after installation of the elastomeric separators, 63.3% of the patients presented spontaneous pain and 70% occlusion pain. After 24 hours, 86.7% of the patients presented spontaneous pain and 100% occlusion pain. After 5 days, 30% of the patients presented spontaneous pain and 60% occlusion pain.

Qamruddin et al. [23]	Numerical Rating Scale	24 hours, for the next 7 days	A single dose of LLLT reduced spontaneous and chewing pain	(1) Peak pain in 24 hours. (2) Significant reduction in the intensity of chewing pain on the third day. (3) Lesser intensity of spontaneous and chewing pain in the LG as compared with the CG on days 1, 2, 3, 4, 5, 6, and 7. (4) Although significantly less, some degree of pain was present even on day 7.	(1) Peak pain in 24 hours. (2) The highest intensity of pain was associated with chewing on the placebo side. (3) Although significantly less, some degree of pain was present even on day 7.

Tortamano et al. [35]	Harazaki + Numeric Rating Scale	Pain start, most painful day, and end of pain	LLLT efficiently controls pain caused by the first archwire	(1) Lower intensity of oral pain compared to the placebo and control groups. (2) Lower intensity of pain on the day of greatest pain compared to the placebo and control groups. (3) No differences found between maxilla and mandible. (4) The pain ceased more quickly in the experimental group (day 3) than in the placebo and control groups. (5) LLLT can control pain from placement of the first archwire. (6) The day of greatest pain was similar for both groups. It occurred between 24 and 48 hours after activation of the orthodontic appliance. (7) The patients in the experimental group generally report oral pain only on touching, while in the control and placebo groups they reported continuous and also chewing pain.	

**Table 4:** GRADE quality evidence.

Outcomes	N. of participants (studies)	Quality of the evidence (GRADE)	Anticipated absolute effects	
			Risk with placebo	Risk difference with pain laser
Spontaneous pain 24 h, elastomeric separators	203 participants (4 RCTs)	*⨁⨁*◯◯ low^a^	—	SMD *0.76 SD lower* (1.19 lower to 0.33 lower)

Spontaneous pain 24 h, archwire placement	60 participants (2 RCTs)	*⨁⨁*◯◯ low^b^	—	SMD *2.09 lower* (4.1 lower to 0.09 lower)

Spontaneous pain 72 h, elastomeric separators	185 participants (3 RCTs)	*⨁⨁*◯◯ low^c^	—	SMD *0.54 lower* (0.91 lower to 0.17 lower)

Spontaneous pain 72 h, archwire placement	20 participants (1 RCT)	*⨁⨁*◯◯ low^d^	—	SMD *1.54 lower* (2.57 lower to 0.51 lower)

Chewing pain 24 h, elastomeric separators	160 participants (3 RCTs)	*⨁⨁*◯◯ low^e^	—	SMD *0.99 lower* (1.28 lower to 0.7 lower)

Chewing pain 72 h, elastomeric separators	160 participants (3 RCTs)	*⨁⨁*◯◯ low^e^	—	SMD *0.68 lower* (1.03 lower to 0.32 lower)

CI, confidence interval; SMD, standardised mean difference; GRADE, working group grades of evidence: high quality: we are very confident that the true effect lies close to that of the estimate of the effect; moderate quality: we are moderately confident in the effect estimate; the true effect is likely to be close to the estimate of the effect, but there is a possibility that it is substantially different; low quality: our confidence in the effect estimate is limited; the true effect may be substantially different from the estimate of the effect; very low quality: we have very little confidence in the effect estimate, the true effect is likely to be substantially different from the estimate of effect; explanations: ^a^the evidence was downgraded by two levels because of very serious concern regarding the risk of bias; one study had high risk in random sequence generation, two studies did not report information regarding allocation concealment, and two studies had a high risk of performance bias; ^b^the evidence was downgraded by one level because one study had high risk regarding selective report and small number of participants; ^c^the evidence was downgraded by two levels because of very serious concern regarding the risk of bias; one study had high risk in random sequence generation, two studies did not report information regarding allocation concealment, and one study had a high risk of performance bias; ^d^the evidence was downgraded by one level because one study had high risk regarding selective report and one level because it is single study (indirectness); ^e^the evidence was downgraded by two levels because of very serious concern regarding the risk of bias; one study did not report information regarding allocation concealment and two studies had a high risk of performance bias.
